# Relación del tratamiento y coste con la ganancia de agudeza visual en la degeneración macular asociada a la edad

**DOI:** 10.23938/ASSN.1052

**Published:** 2023-11-27

**Authors:** Josep-Oriol Casanovas-Marsal, Elisa Viladés Palomar, Francisco de Asís Bartol-Puyal, Rubén Hernández Vian, Luis E. Pablo Júlvez

**Affiliations:** 1 Servicio Aragonés de Salud Hospital Universitario Miguel Servet Servicio de Oftalmología Zaragoza España; 2 Instituto de Investigación Sanitaria de Aragón Zaragoza España; 3 Grupo de Investigación Miguel Servet Oftalmología (GIMSO) España; 4 Universidad de Zaragoza Universidad de Zaragoza Facultad de Medicina Departamento de Cirugía Zaragoza Spain; 5 Instituto Oftalmológico Quirónsalud Zaragoza- Biotech Vision Zaragoza España

**Keywords:** Degeneración macular húmeda, Costos de la atención en salud, Factores de crecimiento endotelial vascular, Análisis costo-beneficio, Wet macular degeneration, Health care costs, Vascular endothelial growth factors, Cost-benefit analysis

## Abstract

**Fundamento:**

Relacionar la ganancia de agudeza visual (AV) con el coste asistencial y de tratamiento con terapia anti-factor de crecimiento endotelial vascular (antiVEGF) en pacientes diagnosticados de degeneración macular asociada a la edad exudativa (DMAE exudativa).

**Pacientes y métodos:**

Estudio observacional, longitudinal, retrospectivo, de pacientes ≥50 años diagnosticados de DMAE exudativa, con AV logMAR entre 0,6 y 0,06, en seguimiento y tratamiento en nuestro hospital de tercer nivel entre el 01/01/2014 y el 31/12/2018.

**Resultados:**

Se incluyeron 778 pacientes, 62,2% mujeres y media de edad 79,83±7,94 años, con 957 ojos con DMAE exudativa. La AV final global (0,65±0,45) aumentó un 3,2% respecto de la inicial. El 60,3% de los ojos recibieron antiVEGF con ranibizumab, el 10,2% con aflibercept y el 29,5% con ambos (mixto). El grupo mixto incrementó significativamente la AV respecto de la inicial, sin diferencias entre grupos. Aunque el seguimiento/tratamiento fue más largo para el grupo mixto, este recibió menos inyecciones antiVEGF y tomografías de coherencia óptica (OCT). El gasto total por año y ojo tratado fue de 1.972,7 €±824,5; los costes fueron mayores para visita, OCT y tratamiento en el grupo de aflibercept, y menores para angiografías con fluoresceína, tratamiento antiVEGF y costes totales en el grupo mixto. La ganancia decimal de AV tuvo un coste de 872 €±1.077,7 sin diferencias significativas entre grupos.

**Conclusiones:**

Los tratamientos antiVEGF con ranibizumab, aflibercept y ambos mantuvieron la AV en pacientes con DMAE exudativa. En general, los costes asistenciales y de tratamiento fueron menores en el grupo que recibió ambos fármacos.

## Abstract (en)

**Background:**

We examined the relationship between visual acuity changes (VA) and the cost of care and treatment with anti-vascular endothelial growth factors (antiVEGF) in patients diagnosed with age-related exudative macular degeneration(exudative AMD).

**Methods:**

Observational, longitudinal, retrospective study of patients ≥50 years of age diagnosed with exudative AMD, with a logMAR VA between 0.6 and 0.06. and 0.06. Follow-up and treatment were done in our tertiary hospital between January 1, 2014 and December 31, 2018.

**Results:**

The study included 778 patients; 62.2% female and mean age 79.83±7.94 years; 957 eyes had exudative AMD. Mean of final VA (0.65±0.45) increasing 3.2% compared to initial values. Ranibizumab was administered to 60.3% of the eyes, aflibercept to 10.2% and ranibizumab + aflibercept (mixed group) to 29.5%. Significant increase in VA was seen in the group with the mixed treatment, with no inter-group differences. Although follow-up/treatment was longer for the mixed group, they received fewer anti-VEGF injections and optical coherence tomography (OCT). The total expenditure per year and treated eye was € 1,972.7±824.5; costs were higher for visit, OCT, and treatment in the aflibercept group, and lower for fluorescein angiography, antiVEGF treatment, and total costs in the mixed group. Decimal VA gain had a cost of € 872±1,077.7 with no significant inter-group differences.

**Conclusion:**

AntiVEGF treatments (ranibizumab, aflibercept, or both) maintained VA in patients with exudative AMD. Overall, care and treatment costs were lower in the group that received both drugs.

## INTRODUCCIÓN

La degeneración macular asociada a la edad (DMAE) exudativa es una enfermedad degenerativa que afecta principalmente a personas mayores de 55 años^1^ y que se caracteriza por presentar neovascularización coroidea, junto con desprendimiento del epitelio pigmentario y neurosensorial, y/o presencia de líquido intrarretiniano^2^^,^^3^ (Anexo I). Los factores desencadenantes son múltiples: alteraciones genéticas, estrés oxidativo, alteraciones del metabolismo lipídico y consumo de alcohol y tabaco^2^^,^^4^. A nivel mundial se estima que 196 millones de personas padecen DMAE exudativa produciendo ceguera a entre 1,34 y 2,42 millones de ellas^1^^,^^5^. Se estima que, en 2040, la prevalencia de DMAE exudativa a nivel mundial aumentará hasta 288 millones de casos^1^^,^^5^^,^^6^. 

En España se diagnosticaron 400.000 personas de DMAE avanzada en 2019, y la incidencia anual es de 16.770 nuevos pacientes/año. Se estima un aumento de la prevalencia de DMAE con el envejecimiento de la población, siendo del 20% en personas mayores de 60 años hasta 2055, produciéndose el mayor incremento en las mayores de 75 años^7^. 

Sin tratamiento, el diagnóstico de DMAE neovascular conlleva un mal pronóstico visual. A los tres años del diagnóstico, más del 40% de los pacientes pierden seis líneas de agudeza visual (AV) LogMar (logaritmo del ángulo mínimo de resolución), y más del 75% tienen una AV igual o menor de 0,1 Snellen^7^.

Inicialmente, el tratamiento de elección para la DMAE era la terapia fotodinámica, registrándose una incidencia de ceguera post tratamiento de 72,5 casos/100.000 personas-año. No obstante, la aparición de nuevos fármacos anti factor de crecimiento endotelial vascular (VEGF), como ranibizumab y aflibercept, administrados mediante inyección intravítrea, hizo que la incidencia disminuyera hasta 8,2 casos/100.000 personas/año^8^. 

En la práctica clínica habitual, la administración de ambos fármacos se realiza con a través de la estrategia terapéutica *treat-and-extend* (T&E), consistente en la dosificación proactiva e individualizada, de modo que el intervalo entre inyecciones se puede extender gradualmente si se mantiene la estabilidad funcional y anatómica, permitiendo reducir la carga de tratamiento^9^^,^^10^. La media de inyecciones administradas durante el primer año es de doce^11^ mientras que en los ensayos clínicos es de diez^12^^-^^14^. Las complicaciones más frecuentes derivadas de la administración de fármacos antiVEGF mediante inyecciones intravítreas son: hemorragia subconjuntival y aumento de presión intraocular, seguidas de inflamación oculares, endoftalmitis y desprendimiento de retina regmatógeno^15^. La endoftalmitis es la complicación más grave y la menos frecuente, con una incidencia del 0,1%^15^^-^^17^. 

La suma total de costes directos e indirectos para el tratamiento de la DMAE exudativa varía según países: el coste en Francia (4.947 €) es superior que en Alemania (3.768 €), por ejemplo, siendo Italia el país con menor coste (3.001 €)^18^.

El objetivo del estudio es relacionar la ganancia de agudeza visual con el coste del acto asistencial y del tratamiento con terapia antiVEGF en pacientes diagnosticados de degeneración macular asociada a la edad exudativa.

## MATERIAL Y MÉTODOS

Estudio observacional, longitudinal y retrospectivo de una cohorte de pacientes diagnosticados de DMAE exudativa en tratamiento y seguimiento en el servicio de Oftalmología del Hospital Universitario Miguel Servet, hospital de tercer nivel de Zaragoza (España), entre el 1 de enero de 2017 y el 31 de diciembre de 2018.

Se incluyeron pacientes con una edad ≥ 50 años, en tratamiento intravítreo con antiVEGF (ranibizumab y/o aflibercept), y con una medición de AV con corrección óptica en escala logMAR entre 0,6 y 0,06. Se excluyeron aquellos pacientes diagnosticados de patología ocular concomitante (glaucoma, neuritis óptica, patología de la retina) y/o de patología sistémica con implicación oftalmológica; en tratamiento previo con terapia fotodinámica (TFD) con verteporfina, esteroides intravítreos o láser; con cualquier episodio de uveítis, retinopatía diabética u otra enfermedad retiniana distinta de DMAE exudativa; con atrofia geográfica o fibrosis subretiniana central en la visita de inicio; con procedimientos oculares previos y/o con tratamiento simultáneo ocular (terapia fotodinámica, administración de corticoides intraoculares, aplicación de láser de itrio, aluminio y granate (YAG), etc.

El estudio fue aprobado por el Comité de Ética e Investigación de la Comunidad Autónoma de Aragón (C.P.-C.I. PI21/434).

Las variables registradas a partir de la historia clínica fueron:

demográficas: edad en el momento del diagnóstico (en años cumplidos) y sexo (mujer, hombre);
clínicas: fecha de diagnóstico de la enfermedad, tiempo desde el diagnóstico (años), ojo patológico (derecho, izquierdo), valoración de la AV inicial mediante logaritmo del ángulo mínimo de resolución (logMAR), valoración de la AV final mediante logMAR;
tratamiento: fecha inicial del tratamiento con antiangiogénicos (ranibizumab o aflibercept o mixto: pacientes que iniciaron el tratamiento con ranibizumab y posteriormente recibieron aflibercept), número de inyecciones de ranibizumab, número de inyecciones de aflibercept;
seguimiento: número de visitas médicas, número de tomografías de coherencia óptica (OCT), número de angiografías con infusión venosa de fluoresceína.

La variable de resultado fue la diferencia en la AV entre la valoración inicial y la final (escala logMAR).

Para el cálculo del coste asistencial se obtuvieron los costes unitarios por acto asistencial proporcionados por la unidad de Contabilidad del Hospital Universitario Miguel Servet de Zaragoza. Los costes establecidos fueron 296,80 € para el fármaco intravítreo, 97,89 € para el acto de la inyección (quirófano, material, etc.), 38,42 € para la visita, 9,96 € para la OCT, y 33,77 € para la angiografía fluoresceínica.

Las variables cualitativas se describieron mediante frecuencias y porcentajes de cada categoría. Las variables cuantitativas se exploraron con la prueba de bondad de ajuste a una distribución normal (test de Shapiro-Will). En caso de seguir una distribución normal se describieron con media y desviación estándar (DE); se compararon entre dos grupos con la prueba t de Student, y entre más de dos grupos con ANOVA de un factor y test *posthoc* de Tuckey (con ajuste de grados de libertad de Welch para la t, y Games-Howell como *posthoc* para el ANOVA si hubo heterogeneidad de varianzas). En caso de no seguir una distribución normal se describieron con la mediana y el rango intercuartílico (RIC) y se compararon entre dos grupos con U de Mann Whitney y entre más de dos con Kuskal-Wallis y test *posthoc* de Dwass-Steel-Critchlow-Fligner; las comparaciones pareadas se analizaron con Wilcoxon. La correlación bivariadas entre las variables cuantitativas se analizó con los coeficientes de correlación de Pearson (r) y de Spearman (rho). Las asociaciones se consideraron significativas si p<0,05. El análisis estadístico de los datos se realizó con el programa estadístico Jamovi versión 2.2.2.

## RESULTADOS

**Figura 1:**
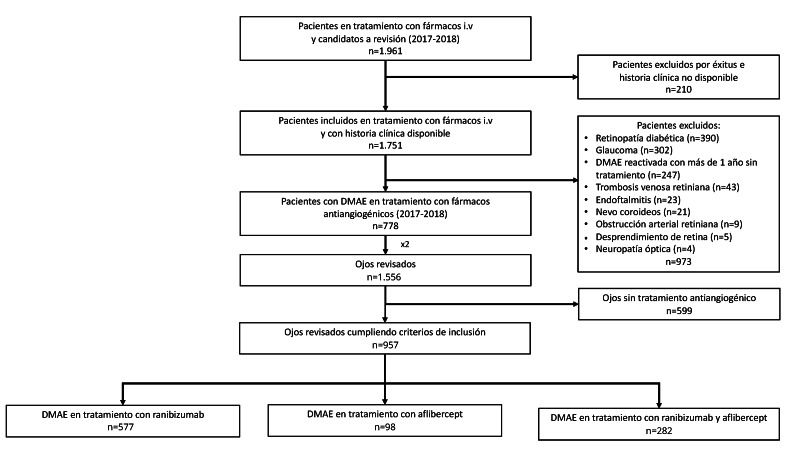
Diagrama de flujo de participación. DMAE: degeneración macular aosiada a la edad exudativa.

Inicialmente, 1.961 pacientes estaban en tratamiento con antiVEGF. Se descartaron 210 por limitaciones en el acceso a sus datos y 973 por cumplir los criterios de exclusión, especialmente retinopatía diabética (n= 390), glaucoma (n=302) y DMAE reactivada con más de un año sin tratamiento (n= 247); algunos pacientes presentaban patología múltiple (Fig. 1). De los 778 pacientes incluidos (1.556 ojos), se excluyeron 599 ojos por no recibir tratamiento antiVEGF. 

Se incluyeron en el estudio 957 ojos correspondientes a 778 pacientes con media de edad al diagnóstico de 79,83 años (DE: 7,94), 62,2% mujeres, que fueron seguidos/tratados durante 2,71 años (DE: 2,43). El 60,3% de los ojos recibieron tratamiento intravítreo con ranibizumab, el 10,2% de aflibercept y el 29,5% con ambos (tratamiento mixto); el 49,8% de los ojos en tratamiento fueron ojo derecho. Se contabilizó una media de 4,32 visitas anuales por ojo tratado (DE: 2,73). El grupo en tratamiento mixto recibió significativamente menos inyecciones y su tiempo de seguimiento y tratamiento fue casi el doble que en los otros dos grupos, con un mayor número de visitas (Tabla 1).

No se hallaron diferencias estadísticamente significativas por sexo para ninguna de las variables estudiadas.

**Tabla 1:** Características y seguimiento de la población de estudio, global y por tipo de tratamiento recibido

Variables	Total	Fármaco (grupo)			Comparación entre grupos
		1: Ranibizumab	2: Aflibercept	3: Mixto	p
					**p *post hoc***
					1-2; 1-3; 2-3
n (%)	957	577 (60,3)	98 (10,2)	282 (29,5)	
*Sexo*, *n (%)*					
Hombre	353 (37)	223 (38,6)	37, (37,8)	93 (33)	0,266^a^
Mujer	604 (63)	354 (61,4)	61 (62,2)	189 (67)	
Edad (años), *media (DE)*	79,8 (7,9)	80,9 (7,5)	79,1 (8,3)	78,0 (8,4)	<0,001^b^ 0,136; <0,001; 0,418
Duración tratamiento (años)*, mediana (RIC)*	2,4 (2,8)	1,4 (2,1)	1,45 (1,7)	3,6 (3,0)	<0,001^c^ 0,788; <0,001; <0,001
*Número al año*					
Visitas de seguimiento, *mediana (RIC)*	4,6 (1,9)	4,6 (2,0)	4,6 (6,0)	4,59 (1,4)	0,704c
Inyecciones*, mediana (RIC)*	5,6 (3,1)	5,7 (3,6)	5,9 (2,4)	5,05 (2,5)	<0,001^c^
					0,112; 0,019; <0,001
OCT, *media (DE)*	5,1 (2,0)	5,1 (2,0)	5,9 (3,00)	4,82 (1,3)	<0,001^b^
					0,005; 0,187; <0,001
AF, *mediana (RIC)*	0,00 (0,34)	0,00 (0,43)	0,00 (0,47)	0,15 (0,27)	0,641^c^ c

a: Chi-cuadrado; b: p de ANOVA seguido de test *post-hoc* Tuckey; c: p de Kruskal Wallis seguido de test *post-hoc* Dwass-Steel-Critchlow-Fligner; AF: angiografía con administración de fluoresceína; DE: desviación estándar; OCT: tomografía de coherencia óptica; RIC: rango intercuartílico.

Antes del tratamiento, la mediana de la AV inicial fue un 29,2% mayor en el grupo que recibió tratamiento solo con ranibizumab. Tras el tratamiento, la media de la AV final total aumentó un 3,2% respecto de la inicial, mientras que la mediana no mostró diferencia. 

Como se puede observar en la Tabla 2, el grupo tratado con ambos fármacos fue el único con una ganancia de AV estadísticamente significativa (media: 0,07 logMAR, DE: 0,33; p=0,002). Aunque de forma no significativa, en los ojos tratados con Aflibercept (grupo 2), la AV disminuyó la ganancia media en 0,04 log MAR (DE: 0,27). En los tres grupos, la ganancia mediana fue cero (Tabla 2).

**Tabla 2:** Agudeza visual en escala logMAR de la población de estudio, global y por tipo de tratamiento recibido

Agudeza visual (logMAR)	Total	Fármaco (grupo)			Comparación entre grupos
		1: Ranibizumab	2: Aflibercept	3: Mixto	p*
					**p *post-hoc***
					1-2; 1-3; 2-3
*Inicial*					
Media (DE)	0,63 (0,43)	0,68 (0,45)	0,58 (0,42)	0,53 (0,35)	<0,001 0,065; <0,001; 0,913
Mediana (RIC)	0,52 (0,49)	0,62 (0,69)	0,48 (0,47)	0,47 (0,39)	
*Final*					
Media (DE)	0,65 (0,45)	0,70 (0,47)	0,53 (0,39)	0,60 (0,41)	0,001
Mediana (RIC)	0,52 (0,52)	0,62 (0,70)	0,40 (0,40)	0,52 (0,40)	0,007; 0,027; 0,266
*Diferencia*					
Media (DE)	0,02 (0,31)	0,01 (0,30)	-0,04 (0,27)	0,07 (0,33)	0,032
Mediana (RIC)	0,00 (0,00)	0,00 (0,00)	0,00 (0,00)	0,00 (0,07)	0,631; 0,074; 0,069
p (Wilcoxon)	0,084	0,575	0,11	0,002	

DE: desviación estándar; logMAR: logaritmo del ángulo mínimo de resolución; RIC: rango intercuartílico; *: p de Kruskal Wallis seguido de test *post-hoc* de Dwass-Steel-Critchlow-Fligner.

Atendiendo a los costes establecidos, el gasto total por año y ojo tratado fue de 1.972,7 € (DE: 824,5; IC 95%: 1.920,5-2.025,0), siendo mayor en el grupo de pacientes tratado con aflibercept que en el de tratamiento mixto. Sin embargo, al analizar la relación entre coste y ganancia de AV, no se hallaron diferencias significativas entre los grupos de estudio, es decir, cada incremento de 0,1 en escala decimal logMAR de AV tiene un coste de 872 € (DE: 1077,7; IC 95%: 720,2-1.024,3) (Tabla 3).

**Tabla 3:** Análisis de los costes de seguimiento y tratamiento, total y por tipo de tratamiento recibido

Coste por año (€)	Total	Fármaco (grupo)			Comparación entre grupos
		1: Ranibizumab	2: Aflibercept	3: Mixto	p*
					**p *post-hoc***
					1-2; 1-3; 2-3
n (%)	957	577 (60,3)	98 (10,2)	282 (29,5)	
*Visitas*					
Media (DE)	203,2 (76,3)	204,4 (77,8)	238,6, (124,6)	190,9 (47,3)	<0,001
Mediana (RIC)	188,69 (57,02)	189,96 (60,59)	210,91 (9123)	182,55 (44,29)	0,017; 0,162; <0,001
*AF*					
Media (DE)	24,49 (27,49)	31,7 (30,49)	37,7 (36,70)	10,60 (7,49)	<0,001
Mediana (RIC)	12,81 (18,79)	19,39 (35,89)	23,41 (33,69)	8,67 (5,73)	0,545; <0,001; <0,001
*OCT*					
Media (DE)	51,05 (19,71)	51,1 (20,02)	59,30 (29,88	48 (12,58)	<0,001
Mediana (RIC)	47,67 (14,75)	47,78 (15,42)	52,47 (20,87)	46,37 (12,04)	0,005; 0,187; <0,001
*Tratamiento*					
Media (DE)	1.744,91 (767,99)	1.780 (842)	1.948 (654)	1.602 (607)	<0,001
Mediana (RIC)	1.720,50 (1.006,70)	1.757 (1.209)	1.860 (813)	1.559 (749)	0,100; 0,009; <0,001
Total** (asistencia y tratamiento)					
Media (DE)	1.972,72 (824,51)	2.012 (905)	2.185 (760)	1.818 (627)	<0,001
Mediana (RIC)	1.929,81 (1.010,19)	1.968 (1199)	2.068 (810)	1.743 (760)	0,171; 0,008; <0,001
Ganancia AV decimal***					
Media (DE)	872 (1.077,71)	836,81 (980.25)	1.841,85 (2.195,04)	736,30 (786,18)	0,126
Mediana (RIC)	488,13 (895,83)	460.88 (856.38)	1.194,17 (1.235,87)	498,64 (786,20)	

AF: angiografías con administración de fluoresceína; AV: agudeza visual; DE: desviación estándar; OCT: tomografía de coherencia óptica; RIC: rango intercuartílico; *: p de Kruskal Wallis seguido de test *post-hoc* de Dwass-Steel-Critchlow-Fligner; **: suma de costes del fármaco intravítreo, del acto de la inyección (quirófano, material, etc.), de la visita médica, de la OCT y de la angiografía fluoresceínica; ***: 0,1 por año, análisis secundario (total, n=193; grupo 1, n=107; grupo 2, n=14; grupo 3, n=72).

## DISCUSIÓN

El presente estudio describe y relaciona la ganancia de AV, así como las visitas anuales de seguimiento, las pruebas complementarias realizadas, y el tratamiento recibido, con los costes asistenciales de los pacientes diagnosticados de degeneración macular exudativa asociada a la edad.

Todos los pacientes fueron atendidos siguiendo las mismas directrices de tratamiento en régimen de T&E. Aproximadamente un 50% de los pacientes comenzaron recibiendo tratamiento con ranibizumab, y un 50% con aflibercept, sin que ninguno de ambos grupos partiera de una peor situación basal.

Los pacientes de nuestro estudio han mantenido la AV inicial durante el tratamiento y seguimiento, con resultados similares a los de estudios previos con mayor media de seguimiento (hasta cinco años^11^ y 10 años^19^). Sin embargo, Westbor y col encontraron que, en pacientes con una agudeza visual de más de 60 letras al diagnóstico, el riesgo de disminuir la AV en menos de 60 letras en el primer y segundo año de tratamiento era del 20%^20^. Características morfológicas de cambio, como nuevas cicatrices foveales, adelgazamiento de las capas de la retina o atrofia foveal, entre otras, son responsables de la pérdida visual irreversible al cabo de cinco años^21^.

El tratamiento intravítreo mediante inyecciones con ranibizumab y aflibercept en la práctica clínica habitual se establece en 12 inyecciones durante el primer año^11^. No obstante, en los ensayos clínicos se establece una media de 10 inyecciones o menos en el primer año de tratamiento y seguimiento^12^^-^^14^, similar a nuestra frecuencia durante el primer año. 

En nuestro estudio, los pacientes a los que se les cambió el tratamiento farmacológico (inicialmente con ranibizumab y después aflibercept, o a la inversa), obtuvieron mejores resultados en relación con la diferencia de AV inicial y final, a diferencia de otro estudio que no observó una mejoría superior en relación a la administración de un fármaco u otro^12^. Esto podría poner de manifiesto la necesidad de prestar atención a la respuesta de cada paciente a cada tipo de anti-VEGF para encontrar el más efectivo en cada caso particular. La valoración del paciente entre 3 y 12 meses tras iniciar el tratamiento para poder realizar el cambio garantizaría que este fuese efectivo^22^^,^^23^, mientras que no se observaron cambios clínicos significativos al realizar el cambio en la reevaluación del paciente tras 12 meses de seguimiento^24^^,^^25^.

Los costes asistenciales generales en nuestro estudio son inferiores a los propuestos por Ruiz Moreno y col^26^ y Cruess y col^27^ en todos los grupos de estudio. 

Aunque los costes totales se relacionen con el beneficio en la ganancia de la agudeza visual de los pacientes con DMAE exudativa, en la mayoría de los estudios esta ganancia implica el mantenimiento de la AV^28^^,^^29^. 

Comparando por tipo de tratamiento, los pacientes tratados con aflibercept tuvieron un coste superior a los otros grupos de fármacos, resultados similares a los de Hernández y col^29^. Se estima que, con el paso del tiempo, el coste asistencial sanitario, disminuirá al recibir un número menor de inyecciones^30^. Sin embargo, se han descrito costes inferiores en los pacientes tratados con aflibercept por recibir menos inyecciones intravítreas^29^, aunque en nuestro estudio fue el grupo que más recibió. 

Como ya se dijo, la suma total de costes directos e indirectos para el tratamiento de la DMAE exudativa puede variar según países de seguimiento y tratamiento: en Italia es de 3.001 €^18^ y en Francia es de 4.947 €. Sin embargo, en países como Suiza el coste mensual puede ascender a una media de 2.000 € por tratamiento con ranibizumab, de 2.500 € con aflibercept y de 2.200 € por tratamiento mixto^31^, cifras muy alejadas del coste medio total de 2.000 € en nuestra población a estudio. El coste de los tratamientos puede ser tan divergente entre países como modelos de sistema sanitario haya.

Los pacientes del grupo mixto también presentaron una mejor relación entre la ganancia de AV y el coste asistencial total, aunque podría deberse a que el seguimiento en este grupo fue significativamente mayor que en los demás. 

Una limitación de este estudio es que los costes, tanto directos como indirectos, se han basado en visitas, administración del tratamiento y pruebas complementarias; es difícil calcular otros gastos como material o el propio uso de las instalaciones sanitarias^28^.

En conclusión, el tratamiento con terapia anti-factor de crecimiento endotelial vascular mantiene la agudeza visual en los pacientes diagnosticados de DMAE. No obstante, el grupo que recibió ranibizumab y aflibercept obtuvo una mejor relación entre la ganancia de agudeza visual y el coste asistencial total.

## Figures and Tables

**Figure:**
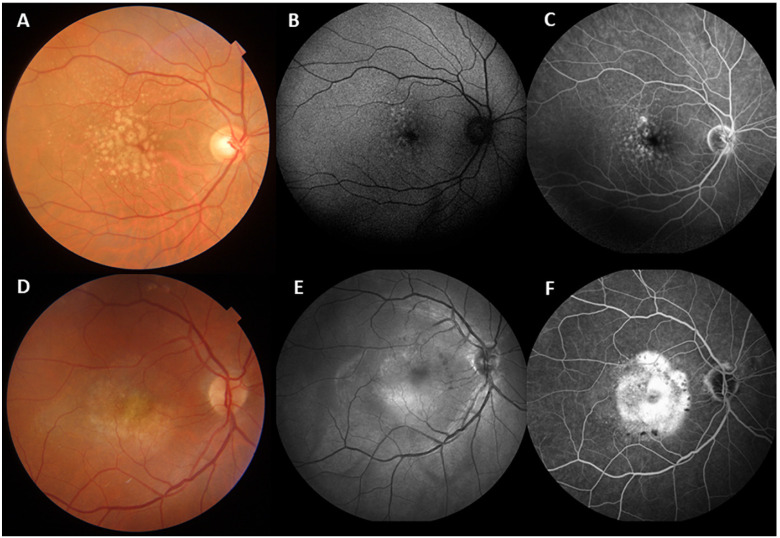
Aspecto morfológico de ojos con degeneración macular asociada a la edad (DMAE)
